# Pit- and trench-forming osteoclasts: a distinction that matters

**DOI:** 10.1038/boneres.2015.32

**Published:** 2015-12-01

**Authors:** Ditte MH Merrild, Dinisha C Pirapaharan, Christina M Andreasen, Per Kjærsgaard-Andersen, Anaïs MJ Møller, Ming Ding, Jean-Marie Delaissé, Kent Søe

**Affiliations:** 1 Department of Clinical Cell Biology, Vejle Hospital/Lillebaelt Hospital, Institute of Regional Health Research, University of Southern Denmark, Vejle, Denmark; 2 Orthopaedic Research Laboratory, Department of Orthopedic Surgery and Traumatology, Odense University Hospital, University of Southern Denmark, Odense C, Denmark; 3 Department of Orthopedic Surgery, Vejle Hospital/Lillebaelt Hospital, Vejle, Denmark

## Abstract

Osteoclasts (OCs) seeded on bone slices either drill round pits or dig long trenches. Whereas pits correspond to intermittent resorption, trenches correspond to continuous and faster resorption and require a distinct assembly of the resorption apparatus. It is unknown whether the distinction between pits and trenches has any biological relevance. Using OCs prepared from different blood donors, we found that female OCs achieved increased resorption mainly through pit formation, whereas male OCs did so through trench formation. Trench formation went along with high collagenolytic activity and high cathepsin K (CatK) expression, thereby allowing deeper demineralization. A specific CatK inhibitor abrogated the generation of trenches, while still allowing the generation of pits. OCs obtained from bone marrow were more prone to generate trenches than those obtained from blood. Scanning electron microscopy of bone surfaces eroded *in vivo* showed trenches and pits of similar size as those made by OCs in culture. We conclude that the distinction between trench- and pit-forming OCs is relevant to the differences among OCs from different skeletal sites, different individuals, including gender, and results from differences in collagenolytic power. This indicates a biological relevance and highlights the importance of discriminating between pits and trenches when assessing resorption.

## Introduction

Bone resorption is performed by osteoclasts (OCs), the only cells known to be capable of resorbing bone.^1^ This property is due to their unique ability to solubilize mineral and collagen, the two main constituents of the bone matrix. Solubilization of mineral is brought about by pumping protons onto the bone surface, thereby generating an acidic environment, whereas collagen degradation is mainly due to activity of cathepsin K (CatK), a cysteine proteinase.^2^ CatK activity and generation of protons have therefore received a lot of attention in the attempt to control the amount of bone degradation and reduce fracture risk.

Importantly, the solubilization of collagen fibers is a more complex event than mineral solubilization, and is by nature a slower process. Furthermore, collagen is not just a substrate to be solubilized by the OC, but also an important antagonist of the polarized resorptive phenotype of the OC.^3^ Therefore, the challenge for the bone resorbing OC is not merely the solubilization of mineral and collagen, but also the synchronization between these two processes. This need for synchronization was particularly stressed in our recent culture study showing how the resorptive behavior of OCs is affected by inhibitors interfering specifically with the rate of either collagenolysis or demineralization.^4^ It was found that when decreasing the rate of collagenolysis versus demineralization, the OCs make preferentially round excavations, sometimes closely aligned. These excavations were interpreted as short-time resorption events separated by migration periods, i.e., intermittent resorption.^5,6^ They represent the well-investigated “pit” resorption mode, and correspond with OCs showing a circular sealing zone, surrounding the so-called resorption zone, with exocytosis of resorption factors occurring at the periphery and endocytosis of collagen fragments at the center (concentric distribution of endocytosis and exocytosis).^7–9^ In contrast, increasing the rate of collagenolysis versus demineralization leads to a higher proportion of long and deep resorption trenches.^4^ They were interpreted as long periods of continuous resorption where the OC moves while resorbing.^10^ They represent the less well-investigated “trench” resorption mode, and correspond with OCs showing a broad crescent of actin at the leading edge of the moving OC, with exocytosis occurring at the inner rim of the actin crescent, followed by endocytosis further to the rear (aligned exocytosis and endocytosis).^8,11^ The trench-resorption mode is expected to lead to faster resorption, since it is not interrupted by migration episodes as it is the case in the pit-resorption mode. Furthermore, trenches represent a shape which favors bone fragility.^12^

From our inhibitor studies,^4^ it may be anticipated that the levels of CatK activity are an important determinant of the resorption mode and of the aggressiveness of the OCs. Because the levels of CatK activity were reported to vary, depending on where the OC originates from,^2,4,13–15^ we hypothesized that OCs from different origins would have a distinct propensity for generating trenches or pits in accordance with their respective collagenolytic levels. Here we analyze this hypothesis and therefore compare the resorption patterns obtained by culturing human OCs of different origins on bone slices. These comparisons include (i) different tissue origins, i.e., OCs generated from CD14^+^ human peripheral blood monocytes versus OCs generated from CD14^+^ human bone marrow (BM) cells; and (ii) interindividual comparisons performed with OCs generated from CD14^+^ human peripheral blood monocytes of different donors and discriminating for gender. The collagenolytic potential of these respective OC populations were evaluated by using one or several of the following approaches: the levels of the collagen fragment, C-terminal cross-linked telopeptide of type 1 collagen (CTX), in the conditioned medium;^16^ the amount of collagen remaining at the bottom of the excavations;^4,17,18^ the response to a specific CatK inhibitor;^4,17,18^ and the expression levels of CatK.^13^ We also included scanning electron microscopy observations of bone surfaces eroded *in vivo*, further verifying the *in vivo* relevance of pits and trenches. Overall, this study aims at evaluating whether the distinction between pit- and trench-making OCs is merely a laboratory peculiarity, or whether it may matter when considering pathophysiological situations.

## Materials and methods

### *In vitro* generation of OCs from human buffy coats

Human CD14^+^ monocytes were isolated from buffy coats (BC) donated by 42 healthy female and 30 male volunteers (approved by the local ethics committee, 2007-0019), and differentiated into mature OCs with macrophage colony-stimulating factor (M-CSF) and receptor activator of nuclear factor kappa-B ligand (RANKL) (R&D Systems, Minneapolis, MN, USA) as previously described.^19,20^ Blood donors are in general between the ages of 18 and 67 years, but their age was not disclosed due to ethical regulation. Informed consent was obtained from all participants. These OCs will be referred to as BC-OCs throughout. For details on the number and gender of the donors used please refer to the figure legends and Supplementary Table S1.

### *In vitro* generation of OCs from human BM

Human BM was obtained from four female and seven male osteoarthritis patients with a mean age of 66 years, undergoing hip replacement (approved by the local ethics committee, 2011-0114). Informed consent was obtained from all participants. For details on the number and gender of the donors used please refer to figure legends and Supplementary Table S1. An X-ray image of the femur was used to eliminate those patients with clear signs of osteoporosis at the site of surgery. The small piece of trabecular bone from the femur removed during surgery was handled as previously described.^21^ The washed out BM was resuspended in minimal essential medium α containing 10% fetal calf serum (FCS; Biological Industries, Kibbutz Beit Haemek, Israel), 25 ng·mL^−1^ M-CSF and RANKL, 2 mmol·L^−1^ L-glutamine (Sigma-Aldrich, Seelze, Germany), 5 ng·mL^−1^ transforming growth factor beta 1 (PeproTech, Rocky Hill, CT, USA), and 10 nmol·L^−1^ dexamethasone (Sigma-Aldrich). Cells were seeded in culture flasks and incubated for 48 h at 37 °C and 5% CO_2_. Those cells that had not attached to the surface were harvested, resuspended in medium, mixed with warm phosphate-buffered saline (PBS) (1:1), and separated on a Ficoll-Paque PLUS gradient. The interphase was collected, washed in PBS, resuspended in medium, seeded in well plates and incubated for 24–72 h. Medium was very gently removed, in order to not remove the semi-attached cells. The cells were then detached with accutase (PAA, Pasching, Austria), washed in medium, and allowed to recover for 30–60 min at 4 °C. The CD14^+^ cells were subsequently isolated as previously described.^19,20^

### Bone resorption assays and analyses

The mature BC-OCs, detached by accutase, and the CD14^+^ BM cells were both seeded on bovine bone slices (IDS Nordic, Herlev, Denmark and Boneslices.com, Jelling, Denmark) at a density of 120 000 cells per bone slice, and cultured in the presence of 10% FCS (Biological Industries), 25 ng·mL^−1^ M-CSF and RANKL, and supplemented with 2 mmol·L^−1^ L-glutamine for the BM cell cultures. When indicated, the specific CatK inhibitor L873724 (a generous gift from Merck, Rahway, NJ, USA) was added to both types of cultures at a concentration of 100 nmol·L^−1^. L873724 was solubilized in dimethyl sulfoxide (Sigma-Aldrich). This solvent was added at the same amount to the controls, resulting in a final concentration of 0.1% (v/v). The number of bone slices used for each condition varied from experiment to experiment between three and eight bone slices per condition.

In order to enable comparisons of resorption characteristics between BC-OC and BM-OC cultures, the duration of the cultures was calibrated according to the length of the trenches. The rationale for doing so is that the length of a trench reflects active resorption time, so that trenches of a similar length reflect similar durations of resorption. Furthermore, length of trenches in cultures of a given duration was consistent. By performing test cultures of different duration, we found that BM-OCs cultured for 4 days and BC-OCs cultured for 3 days generated trenches of similar length.

At culture termination, the cells were removed from the bone slices, resorption events were visualized with toluidine blue (Sigma-Aldrich), and analyzed by light microscopy for the percentage of resorbed surface and resorption depths using a 100-point grid as explained in Boissy *et al*.^19^ and Soe and Delaisse.^20^ The two types of resorption patterns previously described (pits and trenches)^4,20^ were distinguished in the analysis. A pit was defined as a single excavation, circular in appearance, with well-defined edges, and where the ratio between the length and the width of the excavation did not exceed two. A trench was defined as an elongated and continuous excavation, with well-defined edges and at least two times longer than its width. The total resorbed surface was presented as the percentage of the total bone surface, and the trenches, as percentage of trenches per total resorbed surface. The latter enabled a direct comparison between individual experiments irrespective of variations in the total eroded surface area. The maximum resorption depth was measured before and after removal of the organic material, collagen, from the bone surface, and the thickness of the collagen fringe at the bottom of the excavations was calculated as the difference between these two measurements as previously described.^20^ The investigator was blinded during all analyses.

The levels of CTX, in the conditioned medium was measured as previously described^22^ and in accordance with the supplier’s instructions (IDS Nordic, Herlev, Denmark).

### Gene expression analyses

Gene expression analyses were performed on material from experiments with BC-OCs. It was not possible to do this analysis using BM-OCs since it was not possible to obtain enough cells from the same donor to perform both resorption assay and Q-RT-PCR. Mature OCs were generated as described above in the section *in vitro generation of OCs from human buffy coats*), lyzed, RNA purified (Trizol Plus RNA Purification Kit – Invitrogen, Carlsbad, CA, USA), cDNA produced (iScript kit, Bio-Rad, Hercules, CA, USA), and TaqMan Q-RT-PCR in triplicates were performed as previously described.^23^ The TaqMan primer/probes sets used were; GUS: Hs99999908_m1; Abl: Hs00245443_m1; CatK: Hs00166156_m1; MT1-MMP: Hs01037009_g1; and MMP-9: Hs00234579_m1 (Applied Biosystems, Carlsbad, CA, USA). All primer/probe sets were used as instructed by the supplier. Each Q-RT-PCR run was normalized to a cDNA standard curve made each time from the same (randomly chosen) donor. This enabled us to compare between donors and Q-RT-PCR runs. Furthermore, the expression levels of CatK, membrane type 1-matrix metalloproteinase 1 (MT1-MMP), and matrix metalloproteinase 9 (MMP9) were normalized to the average expression level of the house keeping genes, *hGUS* and *hAbl*.

### Fluorescence microscopy

Matured BC-OCs were seeded on cortical bone slices (0.2 mm thick) and allowed to resorb for 3 days as described in the section *Bone resorption assays and analyses*. Cells were fixed with 3.7% formaldehyde for 15 min, washed with PBS, and stained with fluorescently labeled phalloidin for 20 min. Bone slices were washed in PBS, mounted in ProLong Gold containing 4′,6-diamidino-2-phenylindole (Invitrogen), covered with a coverslip, and sealed with clear nail polish. Confocal images were obtained using an Olympus Fluoview FV10i (Olympus Corporation, Shinjuku, Tokyo, Japan) and images were processed using Imaris version 7.6.5 (Bitplan AG, Zurich, Switzerland).

### Scanning electron microscopy

Biopsies were taken from the iliac crest of adult sheep (∼10 years old; approved by Danish Animal Experiments and Inspectorates, 2011/561-1959) from the study described in Andreasen *et al.*^24^ The bone samples were fixed in 4% buffered formaldehyde for 24 h, before transferring to 0.1% buffered formaldehyde. The biopsies were processed according to a previously published procedure^25^ using a Jeol fine coat gold ion sputter JFC-1100. Scanning electron images were made using a LEO 435VP (Zeiss) operated in a secondary electron mode with a 300 V positive bias at the detector. The working distance was 35 mm to assure a large depth of field in the images.

### Statistical analyses

Statistical analyses were performed in GraphPad Prism 4. The types of analyses used are indicated in the figure legends. Significance level was set at *P* < 0.05.

## Results

### Matching *in vitro* and *in vivo* resorption patterns

**Figure 1:**
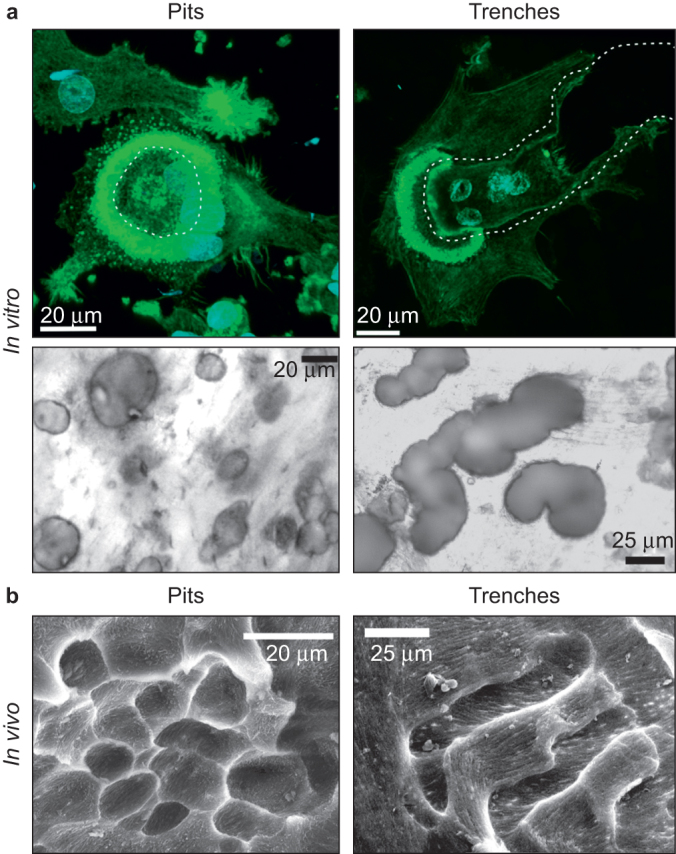
Matching resorption patterns generated by OCs *in vitro* and *in vivo*. (**a**) Pits (left) and trenches (right) generated by OCs *in vitro*, and corresponding actin configurations of these OCs. Bone slices were exposed to the resorptive activity of BC-OCs. Thereafter, they were either fixed, stained for actin and analyzed through confocal fluorescence microscopy (upper a), or were cleared of OCs, stained with toluidine blue and analyzed through ordinary light microscopy (lower a). Herein, we define a “pit” as a round excavation (lower a) generated by an OC showing a circular actin ring (green) which surrounds the pit (dashed line in upper a), as commonly presented. We define a “trench” as a long excavation reflecting a continuous resorption period progressing along the bone surface (lower a). Trenches are generated by OCs showing a broad crescent of actin (green) polarized at the front edge of the elongating trench (dashed line in upper a). (**b**) Pits (left) and trenches (right) generated by OCs *in vivo*. Trabecular bone surfaces from the iliac crest of adult sheep were observed through SEM. The left image shows an area with a resorptive pattern, which closely resembles “pits” observed *in vitro*, while the right image shows an area with clear signs of continuous resorptive activity closely resembling “trenches” observed *in vitro*. Please note that the dimensions of pits and trenches observed *in vitro* and *in vivo* are comparable (scale bars).

Figure 1 illustrates our definition of pits and trenches, both *in vitro* and *in vivo*. Pits are cut perpendicularly to the bone surface by OCs showing a circular actin ring, which surrounds the round hole they are drilling. Trenches are cut parallel to the bone surface, by OCs showing a crescent of actin polarized at the front of the elongating trench they are digging.^11,26^ As investigated by Mulari *et al*.,^26^ this actin distribution is associated with a distinct organization of the resorption machinery in trench-forming OCs, compared to the commonly investigated pit-forming OC.^26^ Trenches are also in line with the mechanism proposed by Stenbeck and Horton^10^ by which OCs simultaneously resorb and progress along the bone surface. Importantly, pits and trenches also occur *in vivo* and are of similar size compared to those observed *in vitro* (Figure 1b), in line with earlier SEM observations.^25,27^

### Bone resorption pattern and collagen degradation characteristics of BM-OCs and BC-OCs

**Figure 2:**
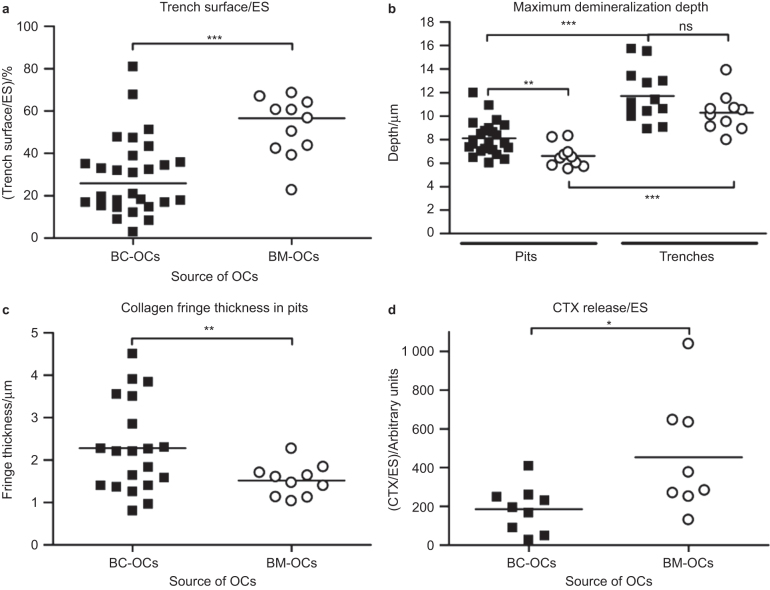
Bone resorption pattern and collagen degradation characteristics of BM-OCs and BC-OCs. BC-OCs and BM-OCs were generated from *n* different donors and cultured on bone slices (3–8 bone slices per condition for each preparation). These slices were then analyzed for: (**a**) percentage of trenches per total eroded surface (BC: *n* = 28, BM: *n* = 11), (**b**) maximum demineralization depth of pits and trenches (BC_pits_: *n* = 23, BC_trenches_: *n* = 13, BM_pits_: *n* = 10, BM_trenches_: *n* =10), (**c**) thickness of collagen fringe in pits only, as defined in the section Materials and Methods (BC: *n* = 20, BM: *n* = 10), (**d**) CTX release per eroded surface (BC: *n* = 9, BM: *n* = 8). The horizontal line in each column indicates the mean (**b**–**d**) or median (**a**). Statistical analyses: Mann–Whitney test (**a**) unpaired *t*-test (**b**) and unpaired *t*-test with Welch correction (**c** and **d**). *P < 0.05; **P < 0.01; ***P < 0.001; ES: eroded surface; ns: not significant.

Whether derived from BM or BC, OCs seeded on bone slices generated both resorption pits and resorption trenches as typical in other reports.^4,11,12,20,28^ The proportion of resorbed surface appearing as trenches (or pits) varied considerably depending on the donor (Figure 2a). However, despite this variation, it appeared that BM-OCs were significantly more likely to generate a high proportion of trenches compared with BC-OCs: the extent of trenches reached more than 40% of the resorbed surface for 10 out of the 11 BM donors, but only for 6 out of the 28 BC donors (Figure 2a). Notably, the difference between the two groups remains highly significant when subdividing them according to the gender of the donors (Supplementary Figure S1).

Of note, trenches were deeper compared with pits (Figure 2b), and in contrast to pits, they showed almost no demineralized collagen at their bottom as reported earlier.^4,20^ This thereby suggests a higher level of collagenolysis in OCs generating trenches than in those generating pits. This held true whether OCs were derived from BC or BM, indicating that greater depths and higher collagenolysis associated with the generation of trenches are independent of the source of OCs. Furthermore, the resorption depth of excavations generated by BC- and BM-OCs were in the same range (Figure 2b). This observation is in line with the general model where OCs resorb perpendicularly to the surface until a certain depth (making pits), and continue resorbing laterally (making trenches).^4,20^ Because trenches are not observed below an average depth of 8 μm, the present data are compatible with the existence of a threshold depth for initiation of trench formation at about a depth of 8 μm.

Since BM-OCs generated a higher proportion of trenches, and since the generation of trenches reflects a high collagenolytic activity, we searched for quantitative indications that BM-OCs might show a higher collagenolytic activity. First, we found that resorption pits generated by the 10 tested preparations of BM-OCs showed less demineralized collagen at their bottom, compared with those generated by most of the preparations of BC-OCs (20 tested in total; Figure 2c). Next, we evaluated the efficacy of collagen degradation by measuring the collagen degradation product, CTX, in the conditioned medium and relating it to the extent of resorbed surface in order to determine the efficiency of collagen degradation during erosion. As shown in Figure 2d, this evaluation showed a higher level of collagen degradation products in the conditioned medium of BM-OCs, compared with those derived from BC.

Thus, taken together, these observations obtained from a large number of independent experiments indicate that a higher propensity to generate resorption trenches goes along with a higher collagenolytic activity during resorption.

### Inhibition of collagen degradation affects the resorption pattern of BM and BC-derived OCs

**Figure 3:**
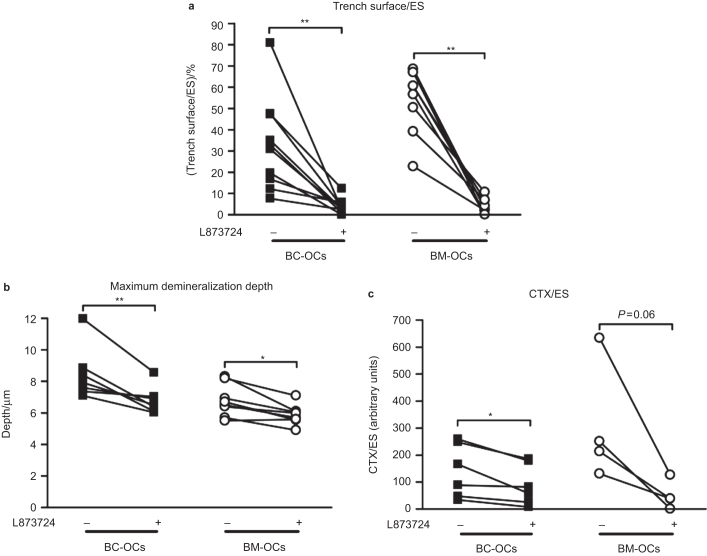
Inhibition of collagen degradation affects the resorption pattern of BM and BC-derived OCs. BC-OCs (black squares) and BM-OCs (open circles) were generated from *n* different donors and cultured in the absence (–) or presence (+) of the specific CatK inhibitor L873724 (100 nmol·L^−1^) on bone slices (3–8 slices per condition and preparation). The bone slices were analyzed for: (**a**) percentage of trenches per total eroded surface (BC: *n* = 10, BM: *n* = 8), (**b**) maximum demineralization depths of pits only (BC: *n* = 8, BM: *n* = 8), and (**c**) CTX release adjusted for eroded surface (BC: *n* = 6, BM: *n* = 4). Statistical analyses: Wilcoxon matched pairs test.

Because the data of the section *Bone resorption pattern and collagen degradation characteristics of BM-OCs and BC-OCs* suggested a relation between generation of trenches and levels of collagenolysis, we investigated whether the formation of trenches would be prevented by inhibiting CatK, which is highly expressed in OCs^29,30^ and is the most potent collagenolytic proteinase known amongst all mammalian proteinases.^31^ Earlier observations on BC-OCs have already shown that prevention of collagen degradation almost abolishes the formation of trenches,^4^ but it had still to be investigated whether this holds true for BM-OCs, which have a higher propensity to generate trenches. Figure 3a shows that whatever the level of trenches in control conditions, inhibition of CatK brings the generation of trenches down to between 0 and 10% of the resorbed surface. This observation shows that CatK-driven collagen degradation is an absolute requirement for the formation of trenches. From a technical point of view, this observation also shows that the inhibitor concentration used in these experiments is sufficient to almost completely prevent this event, whatever its magnitude.

Resorption depth is another excavation-shape parameter commonly believed to be affected by the rate of collagen degradation. Our earlier BC-OC experiments were analyzed without distinguishing between the depths of pits and trenches.^4^ They showed that a general cysteine proteinase inhibitor rendered excavations almost half as deep, in accordance with published knowledge,^2,32–34^ However, only pits are present in inhibitor-treated conditions, whereas both pits and trenches are present in control conditions (Figure 3a). When now taking only pits into account, we found that a specific CatK inhibitor decreased the resorption depths by only 14% in 8 BC-OC preparations, and by 12% in 8 BM-OC preparations (Figure 3b). This shows that the main contribution of CatK to overall excavation depth is by inducing trenches, which prove to be deeper than pits (Figure 2b). However, it is interesting to note that this weak effect (Figure 3b) is enough to bring pit depths below the putative threshold (on average) of about 8 μm for trench formation (Figure 2b). The prevention of trench formation by CatK inhibition may thus result at least in part from a decreased excavation depth.

We also checked whether an endpoint more directly related to collagen degradation was affected by CatK inhibition in these experiments. As expected, CTX levels per resorbed surface were decreased in conditioned media from both BC-OCs and BM-OCs, indicating that overall resorption proceeded with less efficient collagen degradation (Figure 3c).

Taken together, these observations strengthen our previous conclusion that CatK-driven collagen degradation is mandatory for the generation of resorption trenches.^4^ This conclusion was recently confirmed by others,^28^ and is in line with other reports.^35^ Furthermore, the present observations highlight that inhibition of collagen degradation has a bigger impact on the OCs that resorb bone according to the trench pattern, compared to those that resorb bone mostly according to the pit pattern. It is therefore expected that the pharmacological administration of CatK inhibitors may affect bone resorption differently depending on the levels of CatK at different bone sites or in different individuals.

### Comparison of male and female BC-OCs with respect to generation of pits and trenches

**Figure 4:**
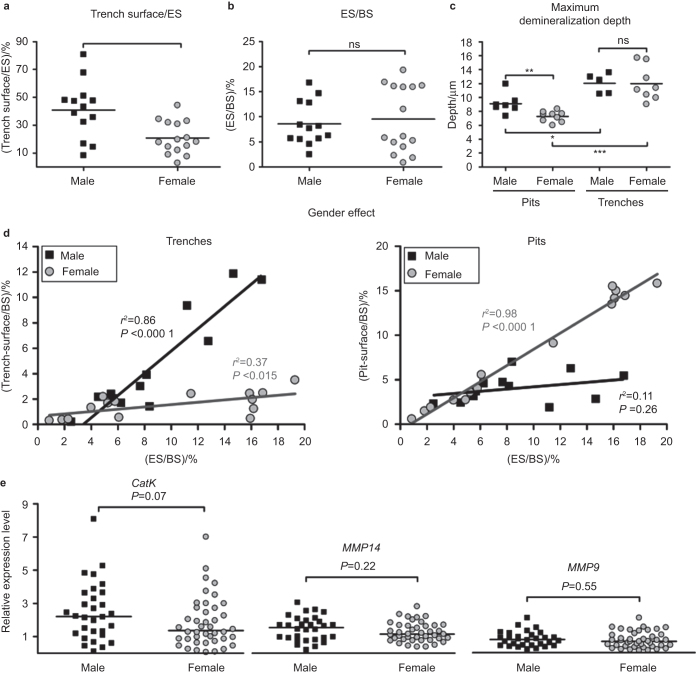
Comparison of male and female BC-OCs with respect to generation of pits and trenches. BC-OCs from male and female donors were generated and cultured on bone slices (3–8 bone slices for each preparation). (**a**) Impact of the gender on the generation of trenches (BC-male: *n* = 13, BC-female: *n* = 15). (**b**) Impact of the gender on the generation of eroded surface (BC-male: *n* = 13, BC-female: *n* = 15). (**c**) Impact of the gender on the maximum resorption depth of pits and trenches (pits BC_male_: *n* = 7, BC_female_: *n* = 9); trenches BC_male_: *n* = 5, BC_female_: *n* = 8). (**d**) Impact of the gender on the relationship between the eroded surface/bone surface and the extent of trench surface/bone surface (left graph) or pit surface/bone surface (right graph) (male: *n* = 13, female: *n* = 15). Correlations and *P* values are indicated in the graphs. (**e**) Gene expression levels of *CatK*, *MMP14*, and *MMP9* in BC-OCs subdivided according to the gender of the donor (male: *n* = 30, female: *n* = 42). Statistical analyses: Mann–Whitney test (**a**, **b**, **c,** and **e**), and linear correlation (Pearson) (**d**). BS: bone surface.

Figure 2a showed the considerable interindividual variation in the proportion of resorbed surface appearing as trenches, depending on the donors. Figure 4a shows that this interindividual variation, at least in part, depends on the gender of the donor. Male BC-OCs erode far more surface as trenches compared with female BC-OCs. This gender effect is strictly on the resorption mode, and is not seen on the total eroded surface (Figure 4b). This indicates a different contribution of trenches and pits to the eroded surface, depending on whether erosion is generated by male or female OCs. In order to get insight in these respective contributions, we plotted trench and pit surface against eroded surface (Figure 4d). These plots show that male OCs achieve increased erosion by generating more trench surface – but not more pit surface – and conversely that female OCs achieve increased erosion by generating more pit surface – but not more trench surface. With respect to resorption depth, Figure 4c shows that trenches are on average around 12 µm deep for both genders. However, the depth of pits generated by male BC-OC resorb is on average 9 µm, which is significantly deeper compared with the average of 7 µm reached by female BC-OCs. These respective depth values further support the hypothesis that a threshold depth of about 8 μm is a prerequisite for trench formation, as mentioned above.

Our results thus clearly show the interest of separately quantifying pits and trenches, and not limiting the measurements to eroded surface. Furthermore they show that one should pay attention to gender when performing osteoclastic resorption experiments.

The reason for the difference between male and female OCs does not relate to differences in fusion potential of male and female OC precursors because OCs from both genders contain the same number of nuclei per OC at the end of the culture (Supplementary Figure S2). However, one may speculate that the reason relates to CatK levels, since a high propensity to generate trenches goes along with high collagenolysis (Figures 2 and 3).^4,20^ Therefore, we compared male and female OCs from a large cohort for their level of CatK expression, as well as for two other proteinases highly expressed in OCs, MMP14 (also called MT1-MMP), and MMP9. The expression level of CatK proved to be highly variable (max/min = 146:1) for both genders, but a clear trend (*P* = 0.07) also suggests that there on average is a higher expression of CatK in OCs generated from male donors than in those generated from female donors (Figure 4e). In contrast, there are no differences between genders in the expression levels of other prominent collagenolytic proteases such as MMP14 (*P* = 0.22) and MMP9 (*P* = 0.55) and also their levels of variation are far less compared to CatK (MMP14: max/min = 16:1; MMP9: 17:1).

## Discussion

The resorption behavior of OCs is routinely investigated on OCs cultured on bone slices, and most effort was devoted to explain the formation of “pits”, i.e., round holes in the bone surface. The commonly accepted model describes alternating periods where the OC attaches to the bone to resorb perpendicularly to the bone surface, and periods where the OC detaches to migrate over the bone surface without resorbing. However, a few reports indicate that cultured OCs are also able to resorb while migrating,^8,10^ thereby generating trenches which reflect long periods of resorption parallel to the bone surface. This “trench” resorption mode did not receive much attention, despite its very contrasting characteristics compared with the “pit” resorption mode (Table 1). The trench mode was recently proposed to involve high rates of collagenolysis versus demineralization, as investigated through pharmacological manipulation.^4^ The present study shows that the distinction between the trench- and pit-resorption mode is relevant to the natural variations found *in vivo* and in human OCs of different origin cultured on bone slices. Furthermore, it shows that this distinction is systematically associated with differences in the levels of collagenolysis or of CatK expression, thus supporting the critical role of CatK in trench formation.

First, we draw the attention on the resemblance between *in vivo* resorption patterns and the pits and trenches generated in culture. Variations in the shape of the excavations made by OCs *in vivo* have been known for a long time,^36^ but they had never been ascribed to possible distinct resorption modes of the OCs, as the existence of distinct resorption modes had not yet been recognized. Of note, Parfitt^37^ inferred from observations on bone sections, that OCs *in vivo* are often likely to resorb bone parallel to the bone surface. Full understanding of *in vivo* bone remodeling may thus require more attention for the poorly investigated trench mode, rather than for the pit mode.

Natural variations between the OC populations generated from different blood donors are well known by bone scientists because they appear as a technical problem rendering experimental reproducibility difficult to achieve when working on human material. However, this variation is usually not addressed, even if it is of potential interest in relation with differences in fracture risk, outcome of bone disease, and response to anti-resorptive treatment among different patients. Herein we extend this natural variation between donors to the prevalence of trenches, indicating that some patients may be subjected to a more aggressive type of resorption than others. A remarkable finding is that male OCs generate more trench surface, not pit surface, when eroding more bone, whereas female OCs generate more pit surface rather than trench surface when eroding more bone. This finding is in line with earlier observations showing a higher resorptive activity in male OCs, as assessed *in vitro.*^38^ Furthermore, serum CTX levels are significantly higher in young healthy adult males compared to young healthy adult females,^39–41^ while serum TRACP5b levels, a marker of OC number, do not appear to be significantly different.^41,42^ This observation may reflect higher CatK activity in OCs of males compared to females also *in vivo*. It is presently not clear how this gender difference may relate to previously reported gender differences in osteoclastic gene expression, but TNF-related apoptosis-inducing ligand may be a factor that deserves further investigation in this aspect.^43^

Natural variations among OCs from different skeletal sites have also been repeatedly reported.^44,45^ Differences between flat and long bone OCs have been particularly highlighted with respect to response of resorption to pharmacological agents and to gene defects.^2,14,15,46^ Of interest in respect with the present study is that long bone OCs are richer in CatK than flat bone OCs. Accordingly, deletion or pharmacological inhibition of CatK affected more drastically resorption of long bones than that of flat bones. In contrast, resorption of flat bones but not that of long bones was slowed down by inhibitors of MMPs, weaker collagenolytic proteinases believed to contribute to collagen degradation in situations where CatK is deficient. However, these earlier studies did not consider whether these differences in collagenolytic pathways affect the resorption mode of the OCs, i.e., whether they affect the resorption pattern. Herein, we show that the stronger collagenolytic power of BM-OCs compared to BC-OCs goes along with a higher likelihood of generating a higher proportion of trenches compared to pits. We show that this great generation of trenches is also completely dependent on CatK activity, since the addition of a specific CatK inhibitor abrogates their generation. In contrast, this CatK inhibitor still allows pit formation. CatK inhibitors thus have a stronger impact on OCs exerting aggressive resorption. The mechanism relating CatK activity to trench formation has been extensively discussed in Soe *et al*.^4^ and Soe and Delaisse.^20^ In addition, it is of interest to note that the unique sub-osteoclastic topography created by high collagenolysis^20^ may lead to stabilizing the actin ring,^47^ thereby allowing continuous resorption.

An interesting issue is whether the prevalence of a given resorption mode is due to different precursor origins showing distinct genetic characteristics, or to the response of the same OCs to differences in the environment or levels of systemic regulators. As discussed elsewhere,^44,45^ there are indications that both reasons may be involved. The importance of genetic characteristics is clearly supported by the present findings that the gender predisposes to the erosion mode, i.e., trenches or pits. Regarding the heterogeneity resulting from environmental conditions, it is of interest that CatK expression and the rate of collagenolysis can to some extent be modulated independently from the rate of demineralization, for example, through glucocorticoids^20^ (see discussion in Refs.^4,20^)

We are aware that this study has some drawbacks due to the mostly retrospective nature of the study. We have not been able to age-match the cohorts, since we did not have an ethical approval to obtain the age of the blood donors. However, in the study of Jevon *et al*.,^38^ it was demonstrated that there was no difference in the extent of resorption by BC-OCs obtained from donors that were younger than 50 years and those that were older. We have not been able to gender-match BC-OCs and BM-OCs. However, Supplementary Figure S1 shows that the difference between BC-OCs and BM-OCs with respect to trench surface per eroded surface is also highly significant when including the differences in the proportion of males and females in the two cohorts into the analysis.

In conclusion, the present study indicates that the classification of cavitations into pits and trenches is relevant to the *in vivo* resorption patterns, to the natural differences between different OC populations, including gender, and to the levels of their collagenolytic power. CatK is mandatory for continued resorption leading to trenches – an information which may be critical when considering CatK inhibitors such as odanacatib as anti-osteoporotic drugs.^48^ The mechanism driving trench-forming OCs deserves attention in future research. However, whatever the mechanism is, these findings already clearly demonstrate the great importance of discriminating between pits and trenches as well as the gender of donors when quantifying erosion in the classical OC resorption assays performed on bone or dentine slices.

## Figures and Tables

**Table 1:** Hallmarks of pit- and trench-forming OCs

Osteoclastic hallmark	Pits	Trenches	References
Movement	NO	YES	[4,8,10,20]
Symmetry of resorption apparatus			
• Actin	Ring	Crescent-like	[8,11]
• Exocytosis	Peripheral	Front side	[8]
• Endocytosis	Central	Back side	[8]
Balanced collagen & mineral clearance	NO (most often)	YES	[4,20] + Present work
Cavitation depth	Shallower (most often)	Deeper	Present work
