# Supplementary figures and images for: Pit- and trench-forming osteoclasts: a distinction that matters
Source: Bone Res. 2015 Dec 1;3:15032–. doi: 10.1038/boneres.2015.32 (PMC4665108; doi:10.1038/boneres.2015.32)

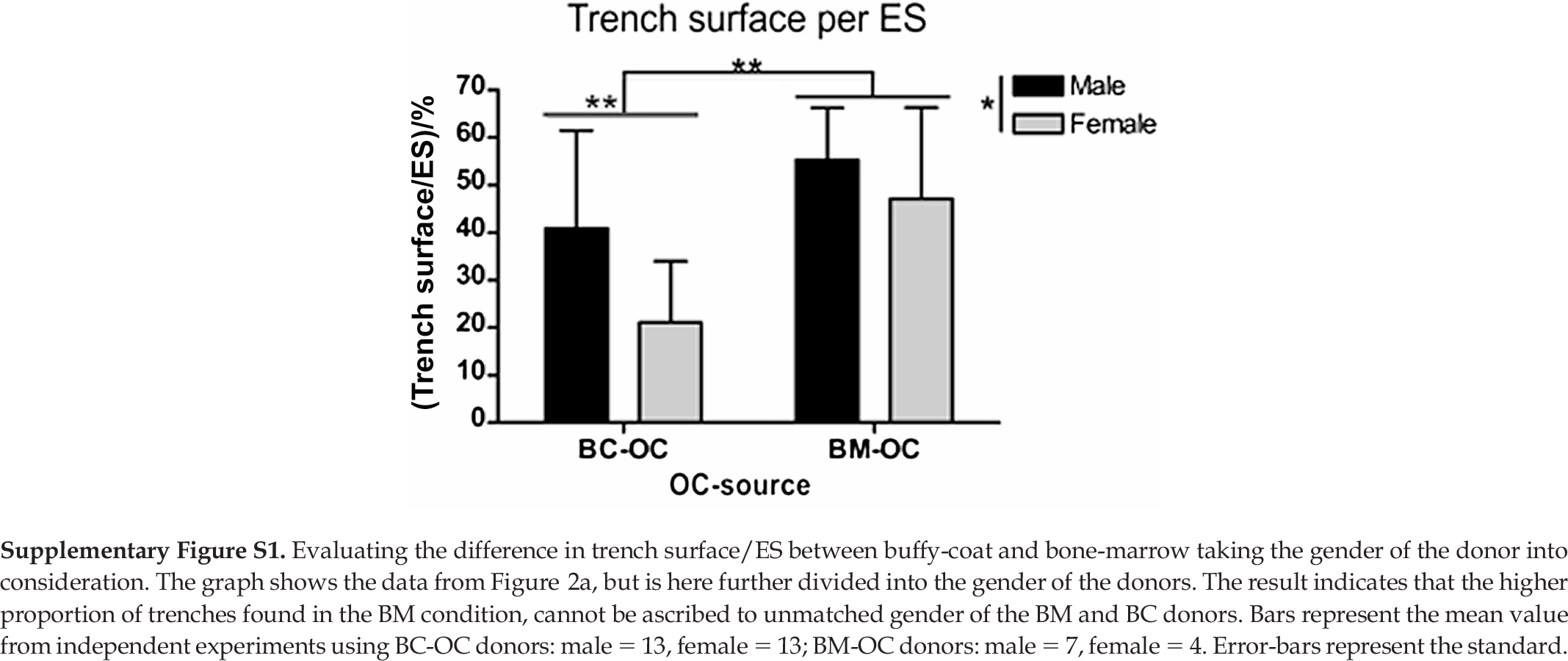

Supplement: Supplementary Figure S1. [file boneres201532-s1.jpg]

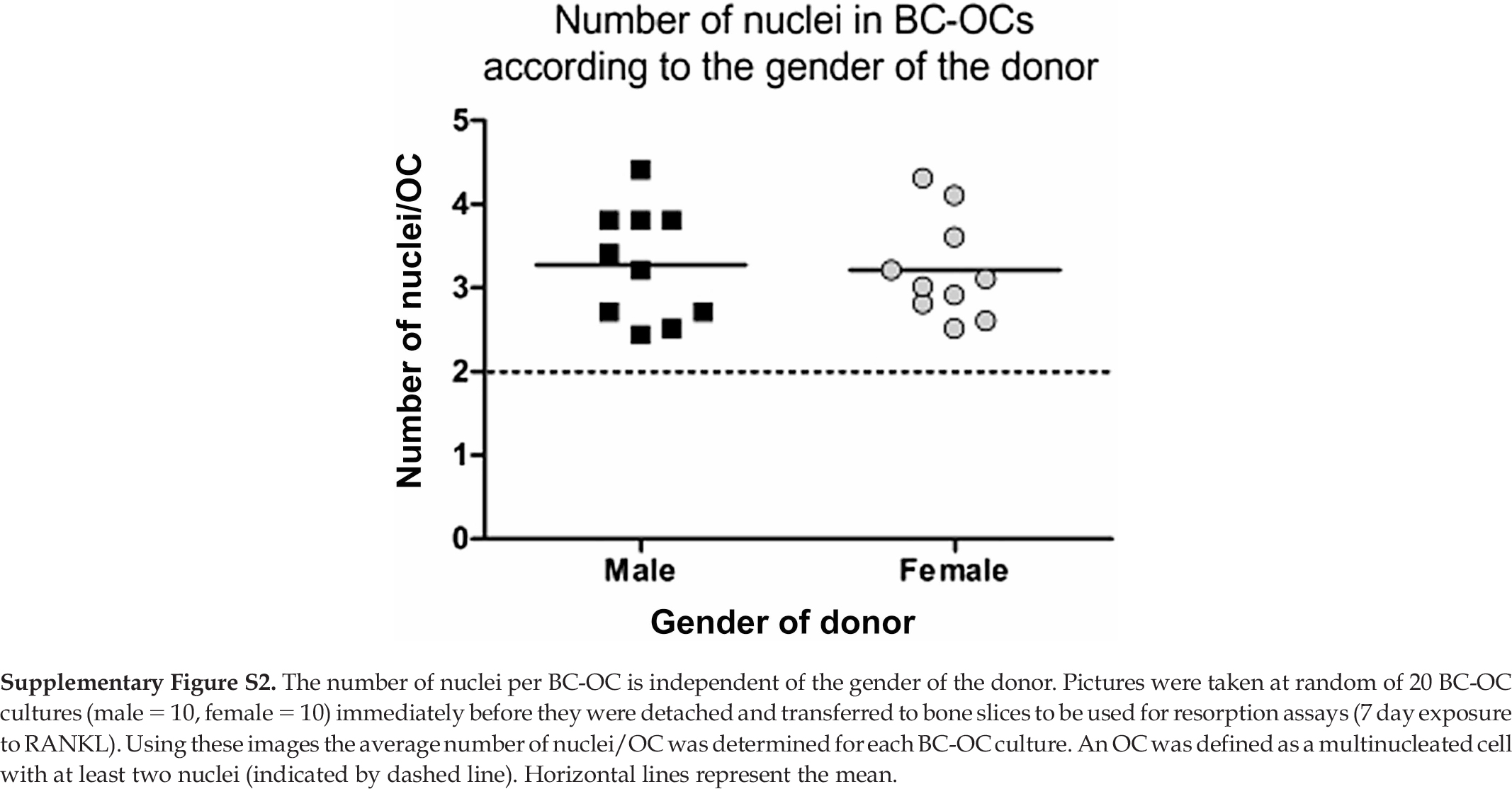

Supplement: Supplementary Figure S2. [file boneres201532-s2.jpg]

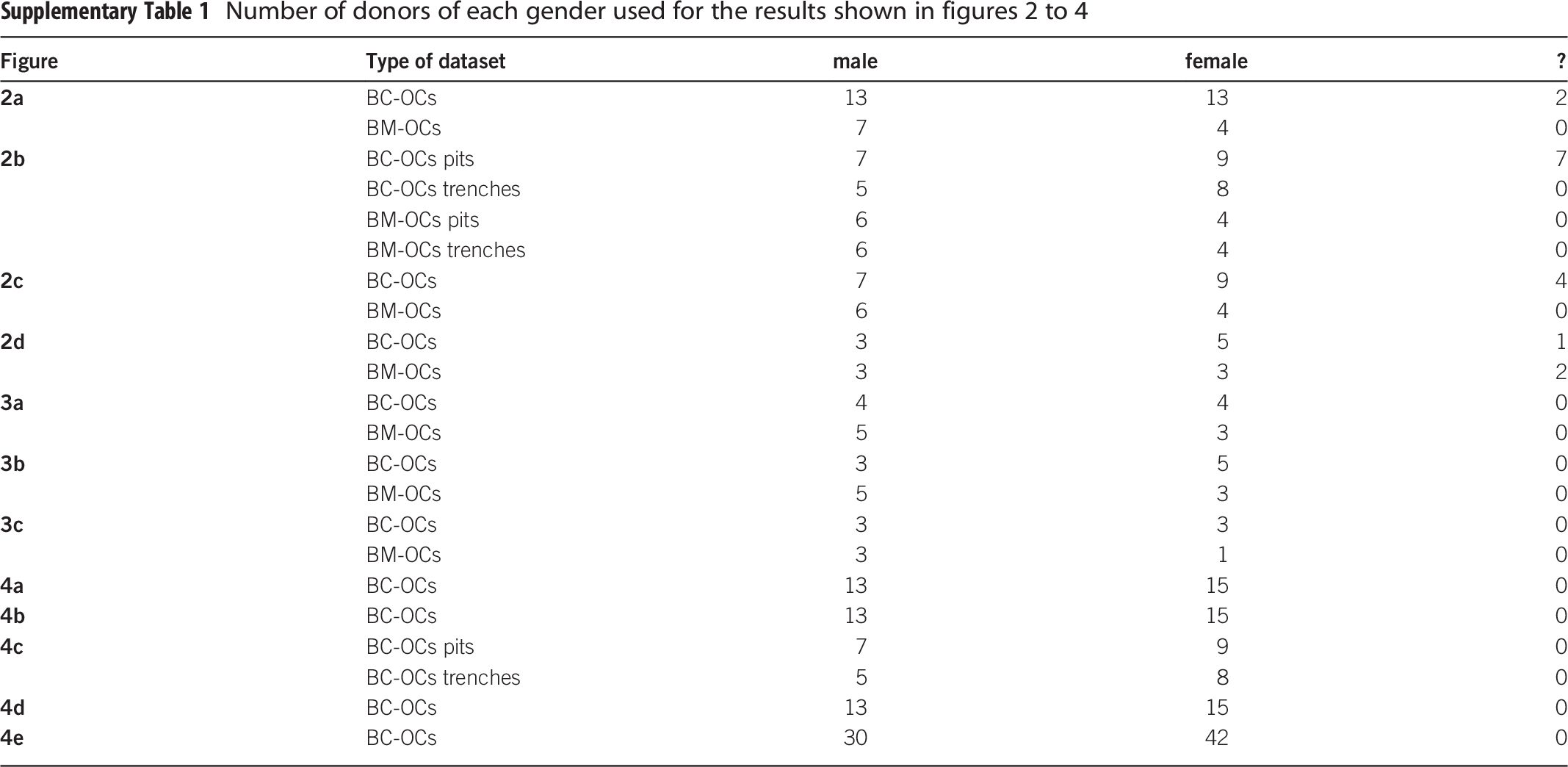

Supplement: Supplementary Table 1. [file boneres201532-s3.jpg]
